# Comparison of serum levels of Tri‐iodothyronine (T3), Thyroxine (T4), and Thyroid‐Stimulating Hormone (TSH) in preeclampsia and normal pregnancy

**Published:** 2012-01

**Authors:** Nayereh Khadem, Hossein Ayatollahi, Fatemeh Vahid Roodsari, Sedigheh Ayati, Ehsan Dalili, Masoud Shahabian, Taraneh Mohajeri, Mohamad Taghi Shakeri

**Affiliations:** 1 Department of Obstetrics and Gynecology, Emam Reza Hospital, Women’s Health Research Center, Mashhad University of Medical Sciences, Mashhad, Iran.; 2 Department of Hematology, Ghaem Hospital, Mashhad University of Medical Sciences, Mashhad, Iran.; 3 Department of Obstetrics and Gynecology, Ghaem Hospital, Mashhad University of Medical Sciences, Mashhad, Iran.; 4 General Physician, Mashhad University of Medical Sciences, Mashhad, Iran.; 5 Department of Medicosocial, Biostatics Unit, Ghaem Hospital, Mashhad University of Medical Sciences, Mashhad, Iran.

**Keywords:** *Tri‐iodothyronine (T3)*, *Thyroxine (T4)*, *Thyroid‐Stimulating Hormone (TSH)*, *Preeclampsia*, *Pregnancy*, *Thyroid*

## Abstract

**Background:** The physiological changes in thyroid gland during pregnancy have been suggested as one of the pathophysiologic causes of preeclampsia.

**Objective:** The aim of this study was comparison of serum levels of Tri‐iodothyronine (T3), Thyroxine (T4), and Thyroid‐Stimulating Hormone (TSH) in preeclampsia and normal pregnancy.

**Materials and Methods:** In this case‐control study, 40 normal pregnant women and 40 cases of preeclampsia in third trimester of pregnancy were evaluated. They were compared for serum levels of Free T3 (FT3), Free T4 (FT4) and TSH. The data was analyzed by SPSS software with the use of t‐student, Chi‐square, Independent sample T-test and Bivariate correlation test. p≤0.05 was considered statistically significant.

**Results:** The mean age was not statistically different between two groups (p=0.297). No significant difference was observed in terms of parity between two groups (p=0.206). Normal pregnant women were not significantly different from preeclampsia cases in the view of FT3 level (1.38 pg/ml vs. 1.41 pg/ml, p=0.803), FT4 level (0.95 pg/ml vs. 0.96 pg/ml, p=0.834) and TSH level (3.51 μIU/ml vs. 3.10 μIU/ml, p=0.386).

**Conclusion:** The findings of the present study do not support the hypothesis that changes in FT3, FT4 and TSH levels could be possible etiology of preeclampsia.

## Introduction

The term preeclampsia describes the development of hypertension ≥140/90 mmHg with proteinuria ≥300mg/24h after 20^th^ week of gestation (1). This disorder is unique to human pregnancy in which numerous genetic immune-logical and environmental factors interact. Therefore, it is a leading cause of maternal and fetal morbidity and mortality throughout the world and still is one of the most complex problems in obstetrics (2). It has long been recognized that maternal thyroid hormone excess or deficiency can influence maternal and fetal outcome at all stages of pregnancy and can interfere with ovulation and fertility (3, 4). During the first trimester, the fetus is reliant on transplacental passage of maternal thyroxine, as the fetal thyroid is not fully functional until about 16^th^ weeks of gestation, whereas thyroid hormone receptors in fetal tissues are functional much earlier (5). 

Although, pregnancy is usually associated with very mild hyperthyroxinemia, women complicated with preeclampsia have high incidence of hypothyroidism that might correlate with the severity of preeclampsia (6). The mechanism of hypothyroidism in preeclampsia has not been identified, but the changes in thyroid function during pregnancy are accounted for by high circulating estrogens (7). There are controversies about the mechanism and clinical significance of low concentration of thyroid hormones in preeclampsia which is related to decreased plasma protein concentrations and increased endothelin level (7). 

Maternal hypothyroidism is the most common disorder of thyroid function in pregnant women and is associated with pregnancy‐induced hypertension, fetal mortality, placental abruption, preterm delivery, and reduced intellectual function in the offspring (8). These outcomes have been associated with both overt hypothyroidism (elevated serum TSH concentration and reduced free T4 concentration), that is found in about 0.2% of pregnant women, as well as subclinical hypothyroidism (elevated serum TSH and free T4 concentration) that is found in about 2.3% of pregnant women (9-11). 

Maternal overt hyperthyroidism (suppressed serum TSH and elevated serum free T3 and T4 concentration) is less common that affects approximately two of 1000 pregnant women (2). Kumar and coworkers in 2003 reported that the level of TSH was increased during first, second and third trimester in all normal pregnant women (12). Larijani *et al* in 2004 reported that serum levels of free T4 and TSH were higher in women with severe preeclampsia compared with mild preeclampsia and normal pregnancy (13). 

The severity and duration of maternal thyroid disease necessary to produce these abnormalities value and timing of therapeutic intervention remain controversial (14, 15). The aim of this study is comparison of serum levels of T3, T4 and TSH in preeclampsia and normal pregnancy.

## Materials and methods

This case‐control, analytic‐descriptive study was performed on 40 women with preeclampsia and 40 normal pregnant women who had referred to Ghaem Hospital related to Mashhad University of Medical Sciences in 2007. The inclusion criteria were all consecutively diagnosed cases of preeclampsia (blood pressure ≥140/90mmHg and proteinuria≥300mg/24h after 20^th^ week of gestation) with gestational age>34 weeks and singleton pregnancy and no history of thyroid disease before and through pregnancy. 

The patients with the history of hypertension, renal disorders, cardio vascular diseases, any metabolic disorder before or during the pregnancy, and history of intake of any medication such as levothyroxine that may affect on thyroid function were excluded from the study. 

Written informed consent was obtained from all patients participating in the study and they were assured about the privacy of the data. The study was approved by the Human Research Ethics Committee of Mashhad University of Medical Sciences. Preeclampsia was defined as mild when blood pressure (BP) exceeded 140/90 mmHg on two more occasions at least 6-h apart and proteinuria exceeded 300 mg/24-h, and as severe when BP was at least 160/110 mmHg and proteinuria exceeded 5 g/24-h (21).

After hospitalization, 3 CC of venous brachial blood samples were obtained from each woman (cases and controls), after the diagnosis was made before the initiation of the antihypertensive treatment, and before delivery (26). Blood pressure values were recorded in a semireclining position by the researcher every 6 h. 

The right arm was used in a roughly horizontal position at heart level. For diastolic blood pressure measurements, both phases (muffling sound and disappearance sound) were recorded. This is very important, since the level measured at phase IV is about 5 to 10 mmHg higher than that measured at phase V. Blood pressure measurements were obtained with random-zero sphygmoma-nometers and were recorded in sitting position (15). 

Thyrotropin (TSH) concentration was measured by a sensitive immunoradiometric (IRMA) method (Kavoshyar, Tehran, Iran), and concentration of free T3 and free T4 by (Immunotech, Marseille, France). All biochemical measurements were performed by RIA. Normal levels of thyroid hormones were as follows: TSH 0.4-4.5 µU/mL, free T4 0.8-2.3ng/dL, free T3 0.13-0.55 ng/dL (25).

All women were followed up through their antenatal, intrapartum and postpartum period. They were especially observed for the development of the symptoms and signs of hypothyroidism.

**Statistical analysis**

Frequency tables were used for description of data, Chi‐square, t‐student test and Bivariate correlation test for analyzing data and logistic regression for controlling the intervention variables. For evaluating two groups, Pearson correlation coefficient test and Independent sample ‘t-test’ were used. p≤0.05 was considered statistically significant.

## Results

A total of 80 women were enrolled in this study. Among them, 40 women with preeclampsia were in case group and 40 healthy pregnant women were in control group. In case group, the mean age of the women was 28±6 yrs and in control group the mean age was 26±5 yrs. No significant difference was observed in the view of mean age, proteinuria and systolic and diastolic blood pressure between two groups (p=0.297) (Table I, Table II).

The mean parity in case group was 1.87±1.45 and in control group was 1.48±1.07. There was no significant difference between two groups in the view of mean of parity (p=0.206) (Table I). In case group the mean T3 level was 1.38±0.74 pg/ml, and in control group it was 1.41±0.39 pg/ml. No significant difference was observed between two groups in the view of T3 level (p=0.803) (Table I). The mean T4 level was 0.95±0.27 pg/ml in case group and 0.96±0.16 pg/ml in control group. 

There was no significant difference between two groups in the view of T4 level (p=0.834) (Table I). The mean TSH level was 3.51±1.84 μIU/ml in case group and 3.10±2.01 μIU/ml in control group. No significant difference was observed between two groups regarding TSH level (p=0.386) (Table I). In both groups, there was no significant difference between T3 level and mean of parity and age, between T4 level and mean of parity and age, between TSH level and mean of parity and age.

**Table I:** Demographic characteristics of the women in both groups

**Statistical index**	**Mean±SD**		**p-value**
**Parameters**	**Case**	**Control**	
Age (year)	28±6	26±5	0.297
Parity	1.87±1.45	1.4±1.07	0.206
FT3 level(pg/ml)	1.38±0.74	1.41±0.39	0.803
FT4 level(pg/ml)	0.95±0.27	0.96±0.16	0.834
TSH level(μIU/ml)	3.51±1.84	3.10±2.01	0.386

**Table II:** Demographic characteristics of the women in both groups

**groups**		**N**	**Mean**	**Std. Deviation**	**Std. Error mean**
Gestational					
	Case	33	35.5758	3.68299	0.64113
	Control	2	38.5000	0.70711	0.50000
High sys- before					
	Case	29	157.5862	20.29341	3.76869
	Control	2	1800000	0.00000	0.00000
High dia- before					
	Case	29	101.8966	13.12126	2.43656
	Control	2	150.0000	7.07107	5.00000
High sys- after					
	Case	29	140.1724	19.47905	3.61717
	Control	2	147.5000	17.67767	12.50000
High dia- after					
	Case	26	89.8077	10.62834	2.08439
	Control	2	82.5000	3.53553	2.50000
24 hours urine protein					
	Case	24	0.9942	1.73162	-
	Control	1	0.0400		-
	Total	25	0.9560	1.70586	-

High dia: high diastolic blood pressure.

High sys: high systolic blood pressure.

## Discussion

Preeclampsia is a serious complication of pregnancy with unknown etiology that may occur at any stage of second or third trimester (16, 17). Although it is defined in terms of hypertension and proteinuria, it can affect other maternal systems, so the presentation and progression of this disease are variable (18, 19). Furthermore, the treatment of this disorder has not significantly changed from 50 years ago so far (20). However, the cause of preeclampsia has remained unknown, but the condition has been reported to be correlated with deficient intravascular production of prostacyclin, a vasodilator, and excessive production of thromboxane, a platelet‐derived vasoconstrictor and stimulant of platelet aggregation (13-15).

The endothelial cell dysfunction plays an important role in the pathogenesis of preeclampsia. Modest decreases in thyroid hormones along with increased TSH level in maternal serum are correlated with severity of preeclampsia and high levels of endothelin (21). Reduced serum concentration of T3 and T4 may also be explained by the faulty estrogen production due to placental dysfunction in preeclampsia. FT4 concentration is not associated with plasma albumin (22). 

Higher levels of FT4 and total T4 (TT4) with lower levels of FT3 and total T3 (TT3) are reported in preeclamptic patients compared to normal pregnant women (23). The titers of FT3 are significantly related to the decreased plasma albumin concentration in preeclampsia (24). It has been suggested that reduced concentration of thyroid hormones in preeclampsia may be due to the loss of protein and protein‐bound hormones in the urine (6).

The results of the present study suggest that the levels of T3, T4 and TSH were not significantly different between preeclampsia and normal pregnancy. A study from Qublan *et al*. performed on 27 severe preeclampsia cases reported that no significant difference was observed in levels of FT4, FT3, and TSH between preeclamptic cases and normal pregnant group with various gestational age subgroups (24). 

Their finding is in accordance with the current study. Kumar and coworkers in 2005 performed the study on 82 pregnant women consecutively admitted with the diagnosis of preeclampsia in the third trimester. Only mean TSH was significantly increased in preeclampsia cases as compared to control subjects (26). 

Their findings suggested that preeclamptic women had higher incidence of biochemical hypothyroidism compared with normotensive pregnant women. This difference in the results may be due to the fact that the time of blood sample was different in the studies; lower levels of T3, T4 along with high level of TSH would be observed at a later stage of preeclampsia.

Some studies reported that serum TT3 and TT4 were significantly decreased and TSH was significantly increased in preeclampsia at third trimester (27). High levels of FT4 and TT4 and low levels of TT3 and FT3 was observed in toxemic patients compared to normal pregnant women (23).

Khandakar and colleagues in 2002 found TT4 level was significantly increased in pregnant women at third trimester of pregnancy, but it was normal in non pregnant women. TT3 and TSH levels didn't increase (28). Moreover, Gulaboglu *et al*. reported that no difference was observed in levels of TSH and FT4 between preeclamptic cases and normal pregnant women, but FT3 level was different between two groups (19). Lao* et al* reported decreased levels of FT4 and increased levels of TSH in preeclampsia (22). 

Vojvodic and associates reviewed medical records of 183 preeclamptic patients; they found a significantly higher incidence of preeclampsia in pregnant women with hyperthyroidism and hypothyroidism (29). The cause of the different results may be difference in study population. Bankowska *et al* reported that thyroid dysfunction was concluded in 78.2% of pregnant women with preeclampsia. They concluded that the thyroid function tests should be performed in all pregnant women with preeclampsia (30). 

The difference in the results of their study with the results of the present study could be various geographical areas, different races, and different diets. Moreover, an abnormally functioning placenta is associated with decreased TBG and higher rates of abortion. Abnormalities in placental function can interfere with oestrogen production that leads to decreased levels of TBG, T3 and T4 (31). Further studies with large population are needed to certainly support the hypothesis that thyroid hormones abnormalities are associated with preeclampsia.

However, with regard to the results of the present study, the measurement of serum levels of T3, T4 and TSG can not be suggested as a criterion for diagnosing preeclampsia. These findings do not support the hypothesis that changes in FT3, FT4 and TSH levels could be possible etiology of preeclampsia.

## Footnote

***This article was extracted from G.P. thesis.***
